# Prognostic Value of Monocarboxylate Transporter 1 Overexpression in Cancer: A Systematic Review

**DOI:** 10.3390/ijms24065141

**Published:** 2023-03-07

**Authors:** Ana Silva, Mónica Costa Cerqueira, Beatriz Rosa, Catarina Sobral, Filipa Pinto-Ribeiro, Marta Freitas Costa, Fátima Baltazar, Julieta Afonso

**Affiliations:** 1 Life and Health Sciences Research Institute (ICVS), School of Medicine, University of Minho, Campus of Gualtar, 4710-057 Braga, Portugal; 2 ICVS/3B’s-PT Government Associate Laboratory, 4710-057 Braga, Portugal

**Keywords:** cancer, Warburg effect, monocarboxylate transporter 1, immunoexpression, prognosis

## Abstract

Energy production by cancer is driven by accelerated glycolysis, independently of oxygen levels, which results in increased lactate production. Lactate is shuttled to and from cancer cells via monocarboxylate transporters (MCTs). MCT1 works both as an importer and an extruder of lactate, being widely studied in recent years and generally associated with a cancer aggressiveness phenotype. The aim of this systematic review was to assess the prognostic value of MCT1 immunoexpression in different malignancies. Study collection was performed by searching nine different databases (PubMed, EMBASE, ScienceDirect, Scopus, Cochrane Library, Web of Science, OVID, TRIP and PsycINFO), using the keywords “cancer”, “Monocarboxylate transporter 1”, “SLC16A1” and “prognosis”. Results showed that MCT1 is an indicator of poor prognosis and decreased survival for cancer patients in sixteen types of malignancies; associations between the transporter’s overexpression and larger tumour sizes, higher disease stage/grade and metastasis occurrence were also frequently observed. Yet, MCT1 overexpression correlated with better outcomes in colorectal cancer, pancreatic ductal adenocarcinoma and non-small cell lung cancer patients. These results support the applicability of MCT1 as a biomarker of prognosis, although larger cohorts would be necessary to validate the overall role of MCT1 as an outcome predictor.

## 1. Introduction

It is well known that the metabolic adjustments involving the Warburg effect largely contribute to an enhanced proliferative capacity and aggressiveness of cancer cells that primarily rely on this phenotype. These adjustments imply a switch from oxidative phosphorylation (OXPHOS) to accelerated glycolysis, regardless of oxygen levels, ultimately resulting in excess lactate production and acidification of the tumour microenvironment (TME) [1,2]. Such a metabolic switch is driven by impaired angiogenesis and oxygenation, as well as modulation of gene expression (up- or downregulation) [2,3]. Monocarboxylate transporters (MCTs) mediate acidification of the extracellular milieu, as these transporters promote lactate extrusion using a proton-linked mechanism; thus, MCTs’ increased expression frequently correlates with cancer cell survival, proliferation, migration, invasion and angiogenesis [4,5,6]. Overexpression of different MCT isoforms, herein focusing on MCT1, has launched several investigations aiming to correlate its inhibition with lower glucose consumption, lactate production, and an impairment in the aforementioned cancer aggressiveness features [4,6].

### 1.1. Glucose Metabolism in Cancer

Although the first observations by Otto Warburg on the field of cancer metabolism were made a century ago [7], it was only in the past decade that this topic gained special interest [8,9,10]. A rapid cancer proliferative index is sustained by a range of metabolic changes, including the adoption of the Warburg effect, which promotes the upregulation of glycolysis in the presence of oxygen [1,2,10,11]. Consequently, the production of high levels of lactate and a decline in the use of pyruvate for tricarboxylic acid (TCA) cycle progression occurs. Glycolysis generates only 2 mol ATP per molecule of glucose (in comparison to 36 mol ATP per molecule of glucose in OXPHOS) [12], but lactate generation is a faster chemical reaction that offers growth benefits to cancer cells, namely cancer cell survival, proliferation, migration, tumour angiogenesis, immunosuppression and resistance to therapy [8,11,13]. These tumorigenesis-associated metabolic alterations are frequently observed in multiple types of malignancies, being introduced as a new hallmark of cancer in 2011 [9].

**Figure 1:**
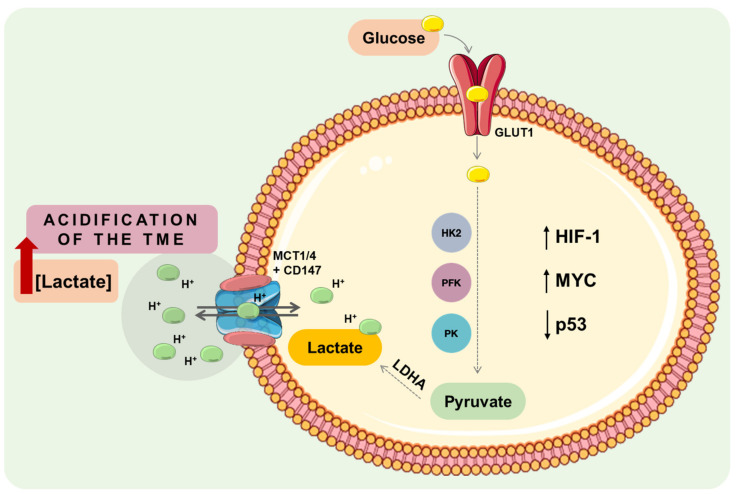
Cancer-associated metabolic alterations occurring with the Warburg phenotype. Intrinsic dysregulation of a range of different genes (oncogene activation, as MYC, or loss of tumour suppressor genes, as p53) leads to the activation of downstream pathways, which, ultimately, promote an increase of glucose consumption, glycolytic flux and lactate production. Altogether, the accumulation of extracellular lactate promotes the acidification of the tumour microenvironment, leading to cancer aggressiveness. CD147, cluster of differentiation 147; GLUT1, glucose transporter 1; HIF-1, hypoxia-inducible factor 1; HK2, hexokinase 2; LDHA, lactate dehydrogenase isoform A; MCT1/4, monocarboxylate transporter 1 or 4; p53, tumour protein 53; PFK, phosphofructokinase; PK, pyruvate kinase; TME, tumour microenvironment.

The metabolic cancer shift occurs in response to a range of genetic alterations combined with the dysregulation of critical transcription factors and/or oncogenic tumour pathways–as MYC, hypoxia-inducible factor-1 alpha (HIF-1α), nuclear factor kappa-light-chain-enhancer of activated B cells (NK-κB), tumour suppressor p53, and phosphatidylinositol 3 Kinase/Akt/Mammalian Target of Rapamycin (PI3K/Akt/mTOR) pathway [14,15,16] (Figure 1).

Deprivation of oxygen diffusion occurs upon tumour growth combined with impaired vascularisation. Thus, in response to these environmental changes, cells activate HIF-1 [17,18]. Additionally, the PI3K/Akt/mTOR pathway can stimulate HIF-1 upregulation. Key players in the glycolytic phenotype are consequently activated, such as glucose transporters (GLUTs), hexokinase 1 and 2 (HK1, HK2), phosphofructokinase (PFK) and pyruvate kinase (PK), lactate dehydrogenase A (LDHA) and MCTs. HK1 and HK2 are responsible for the first committed step of glycolysis (glucose phosphorylation); PFK and PK promote the reduction of glucose to pyruvate; LDHA leads to increased lactate production; and MCTs are the lactate extrusion transporters [13,14,16,18]. In turn, HIF-1 also upregulates pyruvate dehydrogenase kinases (PDKs) to prevent the transition of pyruvate to the TCA cycle. Increased accumulation of lactate at the TME promotes acidification, which stimulates HIF-1 upregulation independently of hypoxia [19]. Cancer metabolism is also influenced by the inactivation of tumour suppressor genes, such as p53, which narrow the use of mitochondrial respiration by downregulating the synthesis of cytochrome c oxidase (SCO2) gene [20] (Figure 1). 

### 1.2. Monocarboxylate Transporters: Key Players in Cancer Aggressiveness

The high rates of energy production through glycolysis in cancer cells result, as mentioned, in increased production of lactate; its accumulation decreases intracellular pH, which would lead to cell death. Lactate is a weak acid, negatively charged at physiological pH, and with no ability to cross the plasma membrane through diffusion. Thus, to avoid cell death, cancer cells rely on several pH regulators, namely carbonic anhydrases, Na^+^/H^+^ exchangers, vacuolar-type H^+^-ATPases, anion exchangers and MCTs [21]. By being proton symporters, MCTs have a dual role in cancer metabolism: to extrude (and also intrude) lactate, thus, supporting the glycolytic phenotype, and to act as pH regulators [6]. MCTs belong to the SLC16 family of genes, which comprehends 14 members. Four of the isoforms (MCT1-4) are known to actively transport monocarboxylates, such as lactate, pyruvate, and butyrate, short-chain fatty acids and ketone bodies by a proton-coupled process [22]. Particularly, MCT1 (SCL16A1) and MCT4 (SCL16A3), both chaperoned by CD147, are strongly associated with cancer aggressiveness, as they were found to be overexpressed in multiple cancers, also correlating with therapy resistance [23,24,25]. MCT1 presents a higher affinity for lactate when compared to MCT4, being an interesting target for therapy. The role of MCT2 is yet to be fully understood regarding its association with cancer progression, but it has been suggested as a possible biomarker in prostate cancer and has a higher affinity for the pyruvate [26]. The role of MCT3 in cancer is still very poorly understood [13].

### 1.3. MCT1 as a Target for Cancer Therapy

MCT1 works in lactate transportation as a bidirectional shuttle with H^+^ mediation [6]. It has been described to be overexpressed in multiple types of tumours, making it an attractive therapeutic target [13]. In bladder and ovarian tumours, MCT1 and its partnership with CD147 have been described to be involved in the cisplatin resistance [27,28], as well as promoting sensitisation to temozolomide in vitro and in vivo in the glioblastoma [4]. 

Inhibition of MCT1 has been attempted using both genetic knockdown and pharmacological inhibitors. Knockdown with siRNA (small interference RNA) led to a decrease in intracellular pH, which inhibited cell proliferation and induced apoptotic cell death in bladder cancer cells [29]. In the same line, knockdown with siRNA and shRNA (short hairpin RNA) inhibited tumour growth, progression and metastasis formation in osteosarcoma in vitro and in vivo [30]. Regarding pharmacological inhibition, one of the first drugs to be tested was α-cyano-4-hydroxycinnamate (CHC), which is not an MCT1-specific inhibitor. Treatment with CHC decreased lactate shuttling, starved glucose-addicted cells and promoted death by necrosis both in vitro and in vivo [31]. This compound inhibited cell growth and lactate uptake in 2D cultures of 4T1 cells, although it did not promote significant alterations regarding tumour volume, weight, and intra-tumour lactate accumulation in 4T1 tumour models [32]. AstraZeneca has developed two compounds that target monocarboxylate transporters: AR-C155858, with inhibitory capacity against MCT1 and MCT2, and AZD3965, a specific MCT1 inhibitor with six-fold less affinity to MCT2, and with no affinity to MCTs 3 and 4. In breast cancer, AR-C155858 was able to decrease cell proliferation in vitro, although it was not able to decrease tumour growth in 4T1 tumour xenografts [33]. This compound was able to decrease lactate exportation and increase cell death in multiple myeloma cells [34]. Treatment with AZD3965 was able to promote cell death involving necrotic processes, successfully decreasing cell proliferation and disrupting lactate exchange upon low MCT4 expression [35]. AZD3965 is currently in a phase I clinical trial for advanced solid tumours and lymphomas in the UK [36]. In a systematic review in which AZD3965′s anticancer effect in mouse models was assessed, the authors concluded that AZD3965 promotes sensitisation to radiation and chemotherapeutic agents, although treatment efficacy seems to be compromised by MCT4 expression since this isoform appears to engage in a compensation mechanism, thus, maintaining lactate shuttling [37].

The promising results on MCT1 inhibition obtained preclinically obviously reflect the frequently reported MCT1 overexpression in clinical samples, as well as its involvement in cancer aggressiveness and poor patient outcome. However, systematic reviews on the prognostic value of MCT1 are lacking. Javaeed and Ghauri conducted a systematic review and meta-analysis, in 2019, on the expression and clinical/prognostic significance of both MCT1 and MCT4. Patients with reduced MCT1 expression exhibited a shorter disease-free survival (HR = 1.48, 95% CI = 1.04–2.10, *p* = 0.03) than patients with high expression; MCT4 stood out as the most important prognostic factor [38]. 

The present systematic review aims to compile existent information regarding the generic prognostic value of MCT1 immunoexpression in different malignancies. We searched nine different databases to analyse 39 studies in which cancer patient samples were used. 

## 2. Materials and Methods

### 2.1. Aim, Study Design and Eligibility Criteria 

The purpose of this systematic review was to analyse distinct survival parameters in cancer patients where MCT1 expression had been evaluated by immunohistochemistry. MCT1 has been the focus of multiple investigations, aiming to achieve a correlation between its expression index and cancer aggressiveness, being thus important to systematically revise the reported clinical studies. 

This systematic review was conducted in accordance with the PRISMA 2020 Statement. Although a review protocol was previously prepared, we did not register it in any registration database.

**Figure 2:**
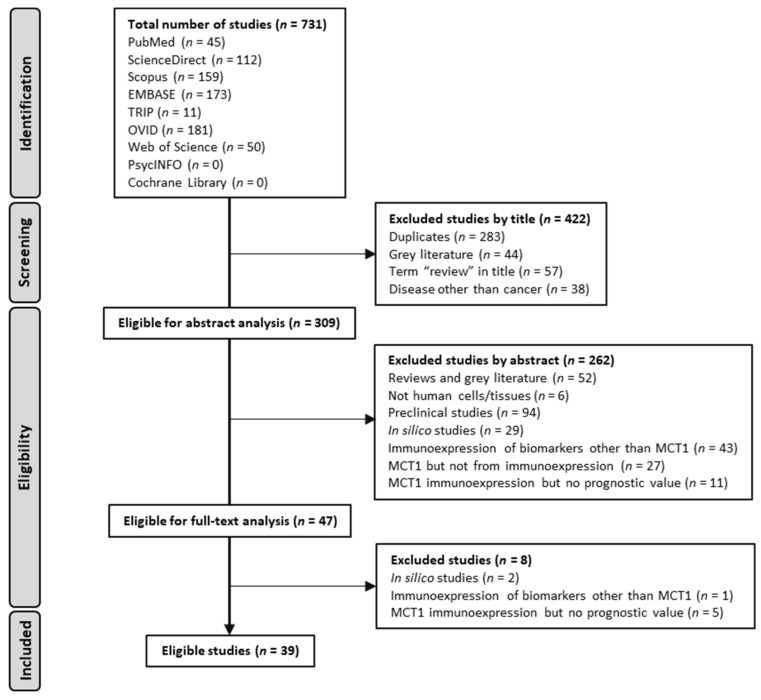
PRISMA 2020 flow diagram for study selection (adapted from [39]).

Eligible studies had to include human cancer patient samples (cohort studies) with an evaluation of MCT1 expression through IHC and its correlation with survival parameters. Furthermore, studies had to be written in the English language. Reports on preclinical studies–including in vitro experiments and in vivo primary studies, studies with MCT1 protein expression retrieved from databases, MCT1 gene expression-only evaluations (mRNA), grey literature, reviews and studies not written in English were excluded from this systematic review (Figure 2).

### 2.2. Search Strategy

A literature search was performed using PubMed, EMBASE, ScienceDirect, Scopus, Cochrane Library, Web of Science, OVID, TRIP and PsycINFO, considering cohort studies published between January 2012 to December 2022. The keywords used as a search strategy were “cancer”, “Monocarboxylate transporter 1”, “SLC16A1”, and “prognosis”, and the search was performed by five independent researchers (A.S., M.C.C., B.R., C.S., and F.P.-R.). No sources other than the above-mentioned were searched, and no authors were contacted.

### 2.3. Study Selection Strategy 

Study selection, as well as full analysis, were performed by five independent researchers (A.S., M.C.C., B.R., C.S., M.F.C. and J.A.). In case of any disagreement, the intervention of the remaining authors was required; those authors were also responsible for review and editing, as well as final approval. Studies were first screened by title and abstract, and those who fulfilled the inclusion criteria were selected for full-text analysis. For each study, the following information was extracted: authors’ names, year of article publication, study design, country, cancer type, number of samples, and distribution by age and gender of patients. Additionally, information regarding antibodies, IHC kit/system, MCT1 signal measurement strategy, expression score (sum or multiplication), and positive cut-off scores were collected. The latter were used to compile information and not for study comparison. Overall study results were also extracted. 

### 2.4. Quality Assessment and Data Synthesis 

Quality assessment of cohort studies was performed by AS and MC using the Critical Appraisal Skills Programme (CASP) Cohort Study Checklist [40]. Studies were evaluated regarding 12 questions, divided into three sections: section A, study validation; section B, study results; and section C, study implications. Questions encompassed aspects such as clarity of the study (1), cohort recruitment (2), outcome measurement accuracy (4), identification of confounding factors (5a) and their consideration in the design and/or analysis of the study (5b), completeness (6a) and duration (6b) of follow-up, description of the results (7), their precision (8), reliability (9), application (10), fitness with other available evidence (11) and practical implications (12). Question 3 (regarding the accuracy of exposure measurements) was not considered for analysis as this is not under the scope of this systematic review. Questions 5 and 6 were divided into the two suggested subgroups (5a and 5b; 6a and 6b), as the authors considered that these aspects should be evaluated separately. All parameters were scored from 0 to 2 points: 0 points given if the parameter was not assessed or if the approach was considered not to be the most adequate; 1 point if the information was lacking or if the approach was reasonable, and 2 points if the information was complete or the approach was valid. These results were translated into a colour scheme: green dots for 2 points, yellow dots for 1 point, and red dots for 0 points. Analysis of the overall quality was based on the sum of all points, being the studies scored as high-quality (≥21 points), moderate quality (14–20 points) and low quality (<13 points). Data were organised in tables and a narrative description was performed.

## 3. Results

### 3.1. Literature Search

The literature search for this systematic review was conducted according to the Preferred Reporting Items for Systematic Reviews and Meta-Analysis (PRISMA) 2020 Statement [39]. Figure 2 summarises the phases of the search. A total of 731 articles were first retrieved, and after screening by title, 309 studies were selected for abstract analysis. From the remaining articles, 262 articles were excluded, as they did not comply with the inclusion criteria. Finally, selected articles were analysed based on their full-text content, resulting in 39 articles (from 2012 to 2022) that were included for further qualitative assessment. 

### 3.2. Characterization and Qualitative Assessment of the Studies 

Throughout all analyses, 39 articles fully fitted the correlation of MCT1 expression with the prognosis of cancer patients. In those studies, samples were collected from the United States of America (n = 2), United Kingdom (n = 3), Norway (n = 2), China (n = 10), Brazil (n = 7), Portugal (n = 5), South Korea (n = 2), Greece (n = 1), Spain (n = 1), Zhejiang (n = 1), Germany (n = 1), Scotland (n = 1), Japan (n = 1) and Finland (n = 2) (Table 1), with a total of 6384 patients. Most of the patients enrolled in the studies were males (66.9%, n = 4475), while 33.1% (n = 2214) were females (125 patients were excluded from this stratification as information on gender was not reported). Cohort numbers (n) ranged from 22 to 560 patient samples. Patient ages ranged between 18 to 98 years, and all samples were collected from adult patients (considering the studies where information on age was available). MCT1 expression was assessed in different cancers: one study in melanoma [41], oral cavity tumours [42], endometrial cancer [43], testicular germ cell tumours [44], soft tissue sarcoma [45], head and neck cancer [46], pancreatic ductal adenocarcinoma (PDAC) [47], small bowel neuroendocrine tumours [48], synovial sarcoma [49], adrenocortical carcinoma [50], small cell lung cancer (SCLC) [35], gastrointestinal stromal tumours (GIST) [51], osteosarcoma [30], malignant pleural mesothelioma [52], Hodgkin lymphoma [53], oropharyngeal squamous cell carcinoma (OSCC) [54] and cancer of unknown primary origin [55]; two studies in prostate adenocarcinoma [56,57], colorectal cancer (CRC) [58,59], clear cell renal cell carcinoma (RCC) [60,61], esophageal squamous cell carcinoma (ESCC) [62,63], non-Hodgkin lymphoma (NHL) [64,65] and gastric cancer [66,67]; three studies in non-small cell lung cancer (NSCLC) [68,69,70] and breast cancer [71,72,73]; and four studies in bladder cancer [27,74,75,76]. 

Concerning the evaluation of MCT1 expression through immunohistochemistry (IHQ) (summarised in Table 2), the most commonly used primary antibody was AB3538P, rabbit polyclonal, from Chemicon International (16 out of the 39 included studies [27,41,42,44,45,50,51,52,55,56,59,60,65,69,74,76]). Nine publications did not report the antibody reference [30,53,54,61,66,67,71,72,75], and two publications used an in-house antibody [35,58]. The most commonly used IHQ kit was R.T.U. VECTASTAIN Elite ABC Kit, Vector Laboratories (14 articles [27,41,42,45,46,47,50,51,56,57,59,65,69,76]). Five studies do not refer to the IHQ kit but refer to the automated system where IHQ was performed [46,47,54,56,69], and ten studies do not refer to any detail regarding the IHQ protocol (kit or system) [30,44,53,58,61,62,63,64,72,75]. 

Most studies did not mention positive or negative controls [30,35,47,49,51,52,53,55,56,58,60,61,62,64,67,68,69,70,71,72,73,74,75]. Two and six studies (respectively) reported the use of negative [43,46] or positive [42,44,48,54,60,66] controls, and eight studies reported using both negative and positive controls [27,41,45,50,57,59,63,65]. For negative controls, replacement or abolishment of primary antibodies were the most reported methods; for positive controls, the most used samples were colon and colorectal carcinoma. MCT1 expression was assessed in the plasma membrane of cancer cells. Cytoplasmic expression was also frequently reported; nuclear expression and expression by stromal cells were additionally mentioned in a few studies, as will be detailed in the next sections.

### 3.3. Global Quality 

To assess the overall quality of the studies, the CASP Checklist (Critical Appraisal Skills Programme) [40] was used. Studies were reviewed according to the twelve questions provided by the checklist and scored from 0 to 26 points. As mentioned in the Materials and Methods section, question number 3 was excluded; questions 5 and 6 were subdivided into sections a and b (5a/5b; 6a/6b). Studies were classified as low quality (<13 points), moderate quality (14–20 points) and high quality (21–26 points). Twenty-two studies were classified as high quality (56.4%), and the remaining seventeen studies were considered to have moderate (15, 38.5%) or low quality (2, 5.1%) (Table 3). Generally, studies lacked a complete patient follow-up scheme. Additionally, most studies could not reproduce existing evidence in total, and implications of the studies regarding clinical practice were not generally foreseen.

### 3.4. Association of MCT1 Expression with Clinicopathological Parameters

Associations among the clinicopathological parameters of the patients and MCT1 expression by cancer cells have been assessed in the large majority of the studies. The statistical significance of the comparisons was mostly assessed by Pearson’s Chi-square or Fisher’s exact test.

An elevated MCT1 expression was associated with older age in the studies by Kim et al. [60] and Martins et al. [59]. No studies reported differences regarding racial groups. Johnson et al. [71] reported a high MCT1 expression in 12% of premenopausal women and 3% of young women with triple-negative breast cancer (TNBC). Zhao et al. [64] showed that female patients with T-cell NHL tended to present higher MCT1 levels than male patients. Opposingly, Tong et al. [68] observed that in female NSCLC patients, MCT1 expression was mostly absent. 

Frequent associations were found between elevated MCT1 protein levels and larger tumours [60,63,71,72], as well as higher disease stage and/or grade [27,41,44,45,50,60,62,63,64,65,66,67,71,73,74]. Of note, Afonso et al., 2016 [76] reported the same associations when MCT1 was expressed by normoxic cancer cells. In another study, Afonso et al. [27] found that such positive correlations were maintained when MCT1 was co-expressed with CD147 [27]; Pértega-Gomes et al. [57] reported that the correlation with the higher stage was only significant when MCT4 was concurrently expressed by stromal cells. Kim et al. [60], Pinheiro et al. [45] and Mikkilineni et al. [53] observed that patients with high MCT1 expression tended to suffer disease recurrence [53] or progression [45,60]. Additionally, associations with vascular invasion [27,44,76] and the presence of proximal (lymph node) and/or distant metastasis [41,44,49,55,60,62,63,74,75] were also commonly noted. In contrast, Sukeda et al. [47] reported that MCT1 expression was inversely associated with regional lymph node metastasis. Additionally, Martins et al. [59] found that although MCT1 expression was increased in CRC primary tumours, a decrease was noted towards lymph nodes and hepatic metastasis [59]. Thirteen studies were not able to establish or did not report any association between MCT1 expression and the clinicopathological parameters [35,42,43,46,48,50,52,54,56,58,61,69,70].

Regarding MCT1 co-expression with other proteins, eleven studies described that MCT1 and CD147 expressions were directly correlated [27,42,43,45,50,51,52,55,59,73,74]. GLUT1 [41,50,55,59], CAIX [41,55], MCT4 [51,55] and CD44 [27] co-expression with MCT1 was also reported. A high degree of correlation among MCT1, CAIX and HIF-1α expressions was observed in the study by Sáenz-de-Santa-María et al. [54]. Curiously, in a cohort of SCLC, 21% of the patients displayed high MCT1 and CAIX expression concomitant with low MCT4 expression [35]. Zhao et al. and Li et al. were able to associate elevated MCT1 expression with higher proliferation index (seen by increased expression of Ki-67) [64,72] and also with higher LDH serum levels [64]. Johnson et al. [71] showed that positive MCT1 expression was associated with negative estrogen receptor (ER) and human epidermal growth factor receptor 2 (HER2) expression, while Li et al. [72] reported the same outcome regarding ER and progesterone receptor (PR) negative tissues.

### 3.5. Prognostic Value of MCT1 Expression

The prognostic value of MCT1 expression (Table 1) was assessed using the Kaplan–Meier method, and the differences were analysed by Log–Rank or Breslow tests. Independent predictors of survival were determined by multivariate analysis using the Cox proportional hazards regression model in most studies.

High MCT1 expression levels were shown to be significantly implicated in a decreased overall survival (OS) of patients with bladder cancer [74,75], RCC [61], melanoma [41], endometrial cancer [43], soft-tissue sarcoma [45], ESCC [62,63], head and neck cancer [46], osteosarcoma [30], SCLC [35], synovial sarcoma [49], adrenocortical carcinoma [50], gastric cancer [67], breast cancer [73] and T-cell NHL [64], remaining as an independent prognostic factor for OS in half of these studies [43,46,49,61,62,63,67,74]. An additional study in bladder cancer [27], and also studies in the GIST [51] and cancer of unknown primary origin [55] reported that OS [27,55] and disease-free survival (DFS) [27,51] were negatively affected when MCT1 and CD147 where co-expressed; this correlation was maintained for decreased OS when co-expression was observed in cisplatin-treated bladder cancer patients [27]. Of note, Afonso et al. [76] observed, in a different report, that cisplatin-treated bladder cancer patients showed decreased OS (near significant associations) when MCT1 expression occurred in normoxic cancer cells, together with MCT4 expression in hypoxic cancer cells and in stromal cells. 

Johnson et al. [71] reported a higher risk of recurrence for MCT1-positive breast cancer patients independent of TNBC status when compared to MCT1-negative patients, in line with Sun et al. [73] and Li et al. [72] and findings, who reported a shorter DFS/recurrence-free survival (RFS) in different cohorts of breast cancer patients [71,72]. Progression-free survival (PFS) was referred to be decreased in breast cancer [71], RCC [60,61], ESCC [62], head and neck cancer [46], T-cell NHL [64] and gastric cancer [67] patients expressing high MCT1 levels; in all of these studies (with the exception of the studies by Zhao et al. [64] and Johnson et al. [71]) MCT1 remained as an independent predictor of cancer progression. MCT1 was also found to be an independent prognostic factor for PFS of Hodgkin lymphoma patients when included in a high metabolic heterogeneity group-high expression levels of TOMM2 and MCT1 in cancer cells and high MCT4 expression in macrophages [53]. Anderson et al. [56] identified MCT1 positivity in prostate cancer cells as a predictor of biochemical failure-free survival, both in uni- and multivariate analysis, only when in association with MCT4 stromal positivity. Sousa-Simões et al. [42] reported that MCT1 expression alone did not have significant implications in OS and disease-free survival (DFS), being a decrease in these survival rates observable when MCT1 positivity, MCT4 positivity and MCT2 negativity were simultaneously associated; this score remained as an independent predictor of overall survival. 

Although most studies reported that elevated MCT1 expression levels are correlated with a worse prognosis, three studies showed the opposite results. Sukeda et al. [47], Eilertsen et al. [69] and Martins et al. [59] observed that elevated MCT1 protein levels were seen in PDAC, in NSCLC and in CRC patients (respectively) who showed increased OS [47,59,69], PFS [47] and disease-specific survival [69] rates. Intriguingly, in the study by Eilertsen et al. [69], such association was inverted when MCT1 expression was observed in the stromal cells. MCT1 nuclear expression was found to be associated with higher overall survival of soft-tissue sarcoma patients in the study by Pinheiro et al. [45]. On the other hand, ten studies reported that MCT1 expression did not have an impact on prognostic parameters [44,48,52,54,57,58,65,66,68,70].

## 4. Discussion

Cancer remains a leading cause of death worldwide, accounting for 9.6 million cancer-related deaths in 2018, according to the World Health Organization (WHO) [77]. The inefficiency of current treatments prompts the search for novel prognostic and treatment-predictive cancer-related biomarkers in order to develop efficient, targeted therapies. Association of MCTs, particularly MCT1, with cancer aggressiveness and poor prognosis has been described in multiple malignancies in past years. Preclinical research on MCT1 has been attempting to disrupt its function as a lactate exporter from cancer cells to the TME. Lactate efflux instigates TME acidification which positively contributes to angiogenesis, migration and treatment resistance [2,6,8,9]. Based on the importance of MCT1 to cancer progression, MCT1-specific inhibitors have been designed and tested, especially in malignancies where MCT1 is overexpressed. However, MCT1 expression in different cancer types does not always produce the same outcome. In this systematic review, studies that highlight the prognostic value of MCT1 expression for cancer patients were analysed. 

From 39 studies evaluated in this review, MCT1 overexpression by cancer cells is associated with a favourable prognosis in NSCLC [69], PDAC [47] and CRC [59] patient cohorts. The results from Eilertsen et al. [69] are in agreement with those reported by Guo et al. [78], which describe that a single nucleotide polymorphism (SNP) of the MCT1 gene (TT genotype of SNP rs1049434) was associated with better OS and RFS of NSCLC patients. Despite this, MCT1 inhibition by AR-C155858 led to an accumulation of intracellular lactate in lung cancer cells in vitro and in xenograft models, also reducing in vivo tumour growth. Yet, the cell lines used in this study were from SCLC; in the same study, MCT1 stood out as a poor prognosis factor for SCLC patients [35]. In the study by Tong et al. [68], the authors did not find any correlation between MCT1 expression and OS, but MCT4 expression was reported to be an independent predictive marker for worse overall prognosis of NSCLC patients; interestingly, these authors stated that patients with MCT1-positive and MCT4-negative tumours (concurrently) are most likely to respond to AZD3965 therapy, as discussed elsewhere [37].

Regarding CRC, besides the results from survival analysis, Martins et al. [59] also found that MCT1 expression decreases from CRC primary tumour towards lymph node and hepatic metastasis. Curiously, an in vitro study has shown that MCT1 is involved in an autophagy-protective mechanism in response to osimertinib (EGFR inhibitor) treatment, in which this compound upregulates MCT1 expression and then activates LKB1/AMPK signalling; thus, revealing a non-canonical role of MCT1, independent of its activity as lactate transporter [79]. On the other hand, MCT4 expression by cancer cells was identified as a poor prognostic factor for CRC patients in both uni- and multivariate analysis in the study by Nakayama et al. [80]. Concerning PDAC, Sukeda et al. [47] observed not only an association between MCT1 overexpression in cancer cells with an extended OS and PFS but also a significant decrease in lymph node metastasis occurrence. An in vitro study showed that genetic or pharmacological inhibition of MCT1 in pancreatic cancer cell lines decreased spheroid growth and invasion but not migration abilities [81]. In another study, although MCT1 expression was restricted to the epithelial compartment and did not associate with prognosis, MCT4 was expressed by both cancer and stromal cells and significantly associated with a worse prognosis [82]. Sukeda et al. [47] found MCT4 stromal expression to be associated with reduced survival. Probably, compensation mechanisms of MCT1-mediated lactate transport by MCT4 function [37], as well as the occurrence of a reverse Warburg effect phenotype upon reprogrammed metabolic requirements–in which MCT1 mediates lactate import to fuel OXPHOS in cancer cells [13]–explain unique metabolic dependencies in these malignancies that are ultimately translated to the clinical setting.

Most of the studies analysed in this review concluded that MCT1 expression by cancer cells could be considered a biomarker for poor survival. In bladder cancer, Choi et al. and Zhang et al. [74,75] observed a significant decrease in patient OS upon elevated MCT1 expression. In the study by Afonso et al. [27] both OS and DFS were decreased upon MCT1 and CD147 dual expression, an association that was maintained (for OS) when considering only cisplatin-treated patients, which highlights the preponderant role of these molecules in mediating chemoresistance. Additionally, this seemed to be correlated with higher disease stage and grade and with the occurrence of both lymph nodes and distant metastasis. CD147 is largely known for its pleiotropic functions in multiple cell types, but its major protumoral action is mediated by its role in chaperoning MCTs [83]. Other studies have reported the role of MCT1 and CD147 in modulating cancer aggressiveness and its importance in the metabolic reprogramming of bladder cancer [84]. The same pattern was detected in melanoma [41] and RCC [60,61]. In both RCC studies herein analysed, MCT1 expression was related to decreased OS and PFS. In silico analysis of TCGA RNA sequence data on the prognostic role of MCT1 in RCC in the study by Kim et al. [60] also revealed the same impact on OS; regarding clinical correlations, in this study, MCT1 expression was found to be associated with older age and larger and highly staged tumours. Cao et al. [61] did not establish any relation to these parameters, but the low number of tissue samples could explain the lack of significance between protein levels and clinicopathological features. Interestingly, for patients undergoing targeted therapy, only decreased PFS, but not OS was observed for RCC patients expressing high levels of MCT1. 

MCT1 also seems to be a prognostic factor for breast cancer patients. Expression of the transporter was frequently observed in large and aggressive tumours and was markedly expressed in patients diagnosed with negative oestrogen and progesterone receptor subtypes. These patients presented shorter OS and D/PFS, as well as an increased recurrence rate. Furthermore, when associated with MCT4, patients presented rapid disease progression and, therefore, a more significant decrease in RFS [71,72,73]. Similar conclusions regarding female cancers were reported for endometrial tumours, where MCT1 was suggested as a marker for worse OS and showed a tendency to be associated with RFS and cancer-specific survival (CSS) [43]. 

MCT1 was found to be an independent prognostic factor for poor OS of gastric cancer patients [67]. Similarly, both Chen et al. [62] and Zheng et al. [63] evaluated the implication of MCT1 expression in ESCC prognosis and concluded that it promotes a significant decrease in patient OS. Additionally, both studies showed a significant correlation between MCT1 expression, increased disease stage and the occurrence of proximal and distant metastasis. In vitro, Chen et al. [62] reported that cell proliferation was significantly compromised, and VEGF (a key player in angiogenesis and cell growth) was downregulated upon MCT1 inhibition. These pre-clinical results are in line with the results from the clinical studies. However, when looking at oesophagal adenocarcinomas, it was described that low MCT1 cytoplasmic expression by cancer cells associated with high tumour stage, presence of lymph node and distant metastasis, and low survival [85], which is in accordance with the above-mentioned studies on NSCLC [69], PDAC [47] and CRC [59], reflecting the specific biological contexts of those malignancies that may be dictating MCT1 role. Regarding head and neck cancer, positive MCT1 staining was associated with both PFS and OS in the study by Leu et al. [46], being this independent of HPV status. Interestingly, for oral cavity cancers, significance regarding decreased OS was only observed in patients with simultaneous overexpression of MCT1 and MCT4 and with little or no expression of MCT2 [42]. In fact, in some types of cancer, MCTs 1 and 4 are associated with a worse prognosis, while MCT2 expression seems to be diminished in aggressive tumours, being inclusively seen as a favourable prognosis marker for hepatocellular cancer [86]. 

In lymphoma patients, analysed studies [53,64] concluded that MCT1 expression associates with worse OS and PFS. MCT1’s specific inhibitor, AZD3965, is currently in a clinical trial for solid tumours and lymphomas [87]. For lymphoma patients, no significant side effects were reported, and patients were observed to have good tolerability to the compound [88]. On the other hand, Anderson et al. reported a significant association between biochemical failure-free survival in prostate adenocarcinoma patients and MCT1 expression by epithelial cells only when in association with MCT4 stromal expression [56]. This has also been observed in other aforementioned studies and is indicative of the metabolic cooperation involving lactate transfer from MCT4-expressing glycolytic stromal cells to MCT1-expressing oxidative cancer cells. This reverse Warburg phenotype, initially described by Sonveaux [31] and Lisanti’s [89,90] groups, has now been expanded to other cancer-associated stromal cells, such as immune and endothelial cells [13]. Either way, MCT-mediated lactate shuttles contribute to cancer aggressiveness, immune tolerance and treatment resistance and are being gradually considered in the search for new anti-cancer therapies [91].

Similarly to the abovementioned malignancies, in other studies in which uncommon types of cancers were analysed–namely GIST [51], osteosarcoma [30], adrenocortical carcinoma [50] and synovial sarcoma [49]–MCT1 expression was significantly associated with a worse prognosis. However, in some cohort studies, we found that MCT1 positivity did not correlate with prognosis, being denominated as non-relevant. For instance, in small bowel neuroendocrine tumours, MCT1 expression showed no prognostic value, although Hiltunen et al. [48] indicated the absence of MCT1 positivity in lymph node metastasis tissues, suggesting that only MCT1-negative cells were prone to develop metastasis. Some confounding factors described by the authors could influence the outcome, such as the low sample size, specifically related to the number of cancer-related deaths; the setting of an optimal cut-off, as the authors observed a decrease in mortality risk associated with an increase in MCT4-positive staining; and the inclusion of only G1 and G2 small bowel neuroendocrine tumour tissues, restraining the correlation of the results with G3 tumours [48]. In the same line, a similar bias was stated by Silva et al. [44]. The authors mentioned that an inadequate cut-off could mask a possible clinical significance associated with MCT1 expression in testicular germ cell tumour tissues. Additionally, Roseweir et al. [58] also pointed out a small sample size together with a relatively small number of CSS events as possible reasons for a lack of MCT1 expression correlation with CSS in CRC. In a distinct setting, Pinheiro et al. [45] observed two divergent correlations between MCT1-positivity and OS, depending on whether the protein is located. The group found that MCT1 nuclear positivity correlated with an increased OS of soft-tissue sarcoma patients; when it was expressed at the plasma membrane of cancer cells, MCT1 correlated with a decrease in OS. The studies by Latif et al. [43] and Afonso et al. [65] also reported MCT1 nuclear expression, although no correlation with survival rates was found. The nuclear expression does not fit with the known function of MCT1 as a plasma membrane lactate transporter, being probably related to a recently described mechanism where lactate drives histone lactylation, an epigenetic mark of the glycolytic switch, and directly modulates gene transcription [92].

Generally, MCT1 overexpression was associated with a worse prognosis for cancer patients. Although most studies were able to correlate protein expression levels and patient outcomes, it is important to note that these results should be analysed carefully. Firstly, some cohorts had very low tissue sample numbers, which, by itself, can have an impact on results. Secondly, it would be important to standardise cut-off values, as this directly influences expression profile analysis. If different cut-offs are applied, then different expression levels will lead to disparities in overall expression patterns. Thirdly, a detailed analysis of MCT1 expression location–membrane or cytoplasm of cancer cells, expression by stromal cells, expression in normoxic versus hypoxic regions–should be performed once different MCT1 locations could potentially unravel different associations with the clinicopathological and prognostic features. Fourthly, we believe these studies should, in the future, be conducted on patients who were widely submitted to therapy. Some studies have already demonstrated that resistance to therapy may be related to MCT1 expression (reviewed in [24]). Therefore, evaluating protein expression in tissues derived from treated patients and comparing them to untreated patients could be of relevance.

It is important to note that we were not able to deepen the statistical analysis of the reported data due to differences in settings, protocols and analysis between works, as often happens in the literature. Nonetheless, one of the advantages of this type of review is the unbiased collection of all the data available as a base for the design of new studies and research lines that produce comparable data between research teams and labs.

## 5. Conclusions

MCT1 expression was significantly associated with unfavourable survival rates in the large majority of the analysed studies (26 out of 39). Overall, MCT1 was identified as a poor prognostic factor in Hodgkin and Non-Hodgkin lymphoma, head and neck cancer, soft tissue sarcoma, renal cell carcinoma, bladder cancer, osteosarcoma, breast cancer, gastrointestinal stromal tumours, oral cavity tumours, prostate adenocarcinoma, melanoma, gastric cancer, oesophagal squamous cell carcinoma, endometrial cancer and adrenocortical carcinoma. Opposingly, in colorectal cancer, pancreatic ductal adenocarcinoma and non-small cell lung cancer, positive expression of MCT1 correlated with increased survival rates. Many studies highlighted that MCT1 seems to play different roles in cancer aggressiveness, being this dependent on cancer cell types. Although MCT1 performs a bi-directional transport of lactate (dependent on pH gradient), this transporter also has a marked affinity for other subtracts, such as pyruvate. Therefore, different prognostic values for this transporter may be explained by the specific role of the transporter within tumours. Moreover, lactate shuttles occurring at the acidic TME and MCT4 compensation mechanisms over MCT1 function also contribute to those differences. Finally, the metabolic heterogeneity of the TME and metabolic flexibility of cancer cells–in which glycolytic cancer cells can return to an oxidative metabolism–clearly add bias to this intricate scenario. 

## Figures and Tables

**Table 1:** The information included in the eligible studies regarding the characterisation of the population and association of MCT1 expression with prognosis.

Reference	Collected Samples (Country)	Cancer Type	n	Age	Sex	Association of MCT1 Expression (Cancer Cells) with Prognosis
de Oliveira et al., 2012 [51]	Brazil and Portugal	Gastrointestinal stromal tumours	Brazil: 51 Portugal: 13	NR	NR	↓DFS when ↑MCT1 + ↑CD147
Choi et al., 2014 [74]	South Korea	Bladder cancer	360	Median: 69 y (range 23–97)	M: 311 (86,4%) F: 49 (13,6%)	↓OS ↓RFS
Eilertsen et al., 2014 [69]	Norway	Non-small cell lung cancer	335	≤65 y: 156 (47%) >65 y: 179 (53%)	M: 253 (76%) F: 82 (24%)	↑OS ↑DSS
Pértega-Gomes et al., 2014 [57]	Portugal	Prostate cancer	480	NR	M: 480 (100%)	No association
Pinheiro et al., 2014 [45]	Brazil	Soft tissue sarcoma	85	≤51 y: 37 (43.5%) >51 y: 48 (56.5%)	M: 52 (61.2%) F: 33 (38.8%)	↓OS; MCT1 nuclear expression: ↑OS
Polanski et al., 2014 [35]	United Kingdom	Small cell lung cancer	58	Median: 61 y (range 35–83)	M: 33 (57%) F: 25 (43%)	↓OS
Zhao et al., 2014 [30]	China	Osteosarcoma	61	NR	NR	↓OS
Afonso et al., 2015 [27]	Portugal	Bladder cancer	114	Median: 70 y (range 41–86)	M: 94 (82.5%) F: 20 (17.5%)	↓DFS + ↓OS when ↑MCT1 + ↑CD147
Andersen et al., 2015 [56]	Norway	Prostate adenocarcinoma	535	≤65 y: 357 (67%) >65 y: 178 (33.0%)	M: 535 (100%)	↓BFFS when stromal ↑MCT4
Kim et al., 2015 [60]	South Korea	Renal cell carcinoma	180	Median: 58 y (range 25–83)	M: 127 (70.6%) F: 53 (29.4%)	↓PFS
Afonso et al., 2016 [76]	Portugal	Bladder cancer	111	Median: 70 y (range: 41–86)	M: 91 (82%) F:20 (18%)	↓OS in cisplatin treated-patients when ↑MCT1 CN + ↑MCT4 CH + ↑MCT4 S *^1^
Martins et al., 2016 [59]	Portugal	Colorectal cancer	500	≤45 y: 23 (4.6%) >45 y: 477 (95.4%)	M: 314 (62.8%) F: 186 (37.2%)	↑OS
Pinheiro et al., 2016 [41]	Brazil	Melanoma	357	Median: 58.3 y (range 25–83)	M: 174 (48.9%) F: 183 (51.1%)	↓OS
Pinheiro et al., 2016 [50]	Brazil	Adrenocortical carcinoma	78	<40.6 y: 34 (43.6%) ≥40.6 y: 44 (56.4%)	M: 17 (21.8%) F:61 (78.2%)	↓OS
Sousa-Simões et al., 2016 [42]	Brazil and Spain	Oral cavity cancer	Brazil: 90 Spain: 45	≤60 y: 69 (50.4%) >60 y: 66 (48.2%)	M: 103 (75.2%) F: 32 (23.4%)	↓OS when ↑MCT1 + ↑MCT4 + ↓MCT2
Giatromanolaki et al., 2017 [70]	Greece	Non-small cell lung cancer	98	Median: 68 y (range 32–81)	M: 86 (88%) F: 12 (12%)	No association
Johnson et al., 2017 [71]	United States	Breast cancer	257	Mean: 57.2 y (range 26.9–97.8)	F: 257 (100%)	↑RR, ↓PFS
Latif et al., 2017 [43]	United Kingdom	Endometrial cancer	90	Median: 67 y (range 57.7–74)	F: 90 (100%)	↓RFS, ↓CSS, ↓OS
Mikkilineni et al., 2017 [53]	United States	Hodgkin lymphoma	22	≤40 y: 14 (64%) >40 y: 8 (36%)	M: 13 (59%) F: 9 (41%)	↓PFS for high metabolic heterogeneity group *^2^
Sáenz-de-Santa-María et al., 2017 [54]	Spain	Oropharyngeal squamous cell carcinoma	249	≤58 y: 134 (53.8%) >58 y: 115 (46.2%)	M: 240 (96.4%) F: 9 (3.6%)	No association
Wang et al., 2017 [67]	China	Gastric cancer	85	≤50 y: 60 (70.6%) >50 y: 25 (24.7%)	M: 52 (61.2%) F: 33 (38.8%)	↓OS, ↓PFS
Cao et al., 2018 [61]	China	Renal cell carcinoma	150	Mean: 56.9 y (TT)/54.6 y (no TT)	M: 84 (56%) F: 66 (44%)	↓OS, ↓PFS
Li et al., 2018 [72]	China	Breast cancer	146	≤64 y: 80.8 (81.6%) >65 y: 28 (19.2%)	F: 146 (100%)	↓RFS
Roseweir et al., 2018 [58]	Scotland	Colorectal cancer	150	≤65 y: 58 (39%) >65: 92 (61%)	M: 83 (55%) F: 67 (45%)	No association
Silva et al., 2018 [44]	Brazil	Testicular germ cell cancer	149	Mean: 32.3 y (range 18–73)	M: 149 (100%)	No association
Zhang et al., 2018 [75]	China	Bladder cancer	124	Median: 65 y (range 30–88)	M: 100 (80.6%) F: 24 (19.4%)	↓OS
Afonso et al., 2019 [65]	Portugal	Non-Hodgkin lymphoma	104	Median: 67 y (range 19–97)	M: 54 (51.9%) F: 50 (48.1%)	No association
Chen et al., 2019 [62]	China	Esophageal squamous cell carcinoma	103	≤60 y: 46 (45%) >60 y: 57 (55%)	M: 68 (66%) F: 35 (34%)	↓OS, ↓PFS
Sukeda et al., 2019 [47]	China	Pancreatic ductal adenocarcinoma	240	<65 y: 86 (36%) ≥65 y: 154 (64%)	M: 154 (64%) F: 86 (36%)	↑OS, ↑PFS
Zheng et al., 2019 [63]	China	Esophageal squamous cell carcinoma	86	≤60 y: 55 (64%) >60 y: 31 (36%)	M: 60 (67.5%) F: 26 (32.5%)	↓OS
Dell’Anno et al., 2020 [52]	United Kingdom	Malignant pleural mesothelioma	135	NR	M: 109 (80.7%) F: 26 (19.3%)	No association
Bonatelli et al., 2021 [55]	Brazil	Cancer of unknown primary origin	118	<59.5 y: 57 (48.3%) ≥59.5 y: 61 (51.7%)	M: 55 (47%) F: 63 (53%)	↓OS when ↑MCT1 + ↑CD147
Eskuri et al., 2021 [66]	Finland	Gastric cancer	560	≤69 y: 283 (49.5%) >69 y: 277 (50.5%	M: 341 (60.9%) F:219 (39.1%)	No association
Leu et al., 2021 [46]	Germany	Head and neck cancer	82	Median: 56.4 y (range 20–88)	M: 67 (81.7%) F: 15 (18.3%)	↓OS , ↓PFS
Tong et al., 2021 [68]	Zhejiang	Non-small cell lung cancer	100	Median: 59 y (range 40–79)	M: 74 (74%) F: 26 (26%)	No association
Yokoo et al., 2021 [49]	Japan	Synovial sarcoma	29	≤40 y: 17 (56.7%) >41 y: 12 (40%)	M: 14 (46.7%) F: 15 (53.3%)	↓OS
Hiltunen et al., 2022 [48]	Finland	Small bowel neuroendocrine cancer	109	Median: 66 y (range 56–72)	M: 60 (55%) F: 49 (45%)	No association
Sun et al., 2022 [73]	China	Breast cancer	137	<50 y: 43 (31.4%) ≥50 y: 94 (68.6%)	F: 137 (100%)	↓OS, ↓DFS
Zhao et al., 2022 [64]	China	Non-Hodgkin lymphoma	38	≤60 y: 22 (57.9%) >60 y: 16 (42.1%)	M: 28 (73.7%) F: 10 (26.3%)	↓OS, ↓PFS

*^1^ Near significant associations; *^2^ High metabolic heterogeneity group, high expression levels of TOMM2 and MCT1 in cancer cells, and high MCT4 expression in macrophages. ↓, decreased; ↑, increased; BFFS, biochemical failure-free survival; CN, normoxic cancer cells; CH, hypoxic cancer cells; CSS, cancer-specific survival; DSS, disease-specific survival; F, female; M, male; n, patient number; NR, not reported; OS, overall survival; PFS, progression-free survival; RFS, recurrence-free survival; RR, recurrence rate; S, stromal cells; TT, targeted therapy; y, years.

**Table 2:** Immunohistochemistry methodology of eligible articles.

Reference	Primary Antibody	Immunohistochemistry Kit/System	Measurement of MCT1 Expression	Positive Cut-Off
de Oliveira et al., 2012 [51]	AB3538P, rabbit polyclonal, Chemicon International	R.T.U. VECTASTAIN Elite ABC Kit, Vector Laboratories	I + E	≥3
Choi et al., 2014 [74]	AB3538P, rabbit polyclonal, Chemicon International	ChemMate EnVision Kit (Dako)	I + MC	>5
Eilertsen et al., 2014 [69]	AB3538P, rabbit polyclonal, Chemicon International	Ventana BenchMark XT (Ventana Medical Systems Inc.)	E	>1.5
Pértega-Gomes et al., 2014 [57]	sc-50329, mouse monoclonal, Santa Cruz Biotechnology	R.T.U. VECTASTAIN Elite ABC Kit, Vector Laboratories	I + E	≥4
Pinheiro et al., 2014 [45]	AB3538P, rabbit polyclonal, Chemicon International	R.T.U. VECTASTAIN Elite ABC Kit, Vector Laboratories	I + E	≥3
Polanski et al., 2014 [35]	Produced in house	Envision Kit (Dako)	I × E	Mean
Zhao et al., 2014 [30]	NR, Millipore	NR	I + E	≥3
Afonso et al., 2015 [27]	AB3538P, rabbit polyclonal, Chemicon International	R.T.U. VECTASTAIN Elite ABC Kit, Vector Laboratories	I + E	≥4
Andersen et al., 2015 [56]	AB3538P, rabbit polyclonal, Chemicon International	Ventana BenchMark XT (Ventana Medical Systems Inc.)	E	>2
Kim et al., 2015 [60]	AB3538P, rabbit polyclonal, Chemicon International	Bond^TM^ Polymer Refine Detection kit	I + MC	>15
Afonso et al., 2016 [76]	AB3538P, rabbit polyclonal, Chemicon International	R.T.U. VECTASTAIN Elite ABC Kit, Vector Laboratories	I + E	≥3
Martins et al., 2016 [59]	AB3538P, rabbit polyclonal, Chemicon International	R.T.U. VECTASTAIN Elite ABC Kit, Vector Laboratories	I + E	≥3
Pinheiro et al., 2016 [41]	AB3538P, rabbit polyclonal, Chemicon International	R.T.U. VECTASTAIN Elite ABC Kit, Vector Laboratories	I + E	≥3
Pinheiro et al., 2016 [50]	AB3538P, rabbit polyclonal, Chemicon International	R.T.U. VECTASTAIN Elite ABC Kit, Vector Laboratories	I + E	≥3
Sousa-Simões et al., 2016 [42]	AB3538P, rabbit polyclonal, Chemicon International	R.T.U. VECTASTAIN Elite ABC Kit, Vector Laboratories	I + E	≥4
Giatromanolaki et al., 2017 [70]	AB85021, rabbit polyclonal, Chemicon International	Thermo-kit Ultravision, Quanto-HRP kit	E	>50%
Johnson et al., 2017 [71]	NR	Ventana Discovery ULTRA	I	≥2
Latif et al., 2017 [43]	sc-365501, mouse monoclonal, Santa Cruz Biotechnology	Leica BOND_MAX and Bond Polymer Refine Detection Kit	I	≥200
Mikkilineni et al., 2017 [53]	NR	NR	I + E	0–2
Sáenz-de-Santa-María et al., 2017 [54]	NR, Abcam	Dako Autostainer Plus	I × E	Median
Wang et al., 2017 [67]	NR	Dako Envision System	I × E	3–12
Cao et al., 2018 [61]	NR, Abcam	NR	I	>2
Li et al., 2018 [72]	NR	NR	I × E	NR
Roseweir et al., 2018 [58]	Produced in house	NR	I × %E	0–300
Silva et al., 2018 [44]	AB3538P, rabbit polyclonal, Chemicon International	NR	I + E	≥6
Zhang et al., 2018 [75]	NR, Abcam	NR	I + E	≥2
Afonso et al., 2019 [65]	AB3538P, rabbit polyclonal, Chemicon International	R.T.U. VECTASTAIN Elite ABC Kit, Vector Laboratories	I + E	≥3
Chen et al., 2019 [62]	20139-1-AP, rabbit polyclonal, ProteinTech Group Inc.	NR	I + E	≥2
Sukeda et al., 2019 [47]	sc-365501 Mouse monoclonal, Santa Cruz Biotechnology	Ventana BenchMark XT (Ventana Medical Systems Inc.)	I + E	≥1
Zheng et al., 2019 [63]	20139-1-AP, rabbit polyclonal, ProteinTech Group Inc.	NR	I × E	≥9
Dell’Anno et al., 2020 [52]	AB3538P, rabbit polyclonal, Chemicon International	Dako EnVision^TM^ FLEX Target Retrieval	I	NR
Bonatelli et al., 2021 [55]	AB3538P, rabbit polyclonal, Chemicon International	Ultra Vision ONE Detection System: HRP Polymer, Lab Vision Corp.	I + E	≥3
Eskuri et al., 2021 [66]	NR, Santa Cruz Biotechnology	Dako EnVision^TM^ FLEX Target Retrieval	I + E	>150
Leu et al., 2021 [46]	sc-50324, rabbit polyclonal, Santa Cruz Biotechnology	Ventana BenchMark XT (Ventana Medical Systems Inc.)	I × E	0–300
Tong et al., 2021 [68]	AB238825, rabbit polyclonal, Chemicon International	Dako EnVision^TM^ FLEX Target Retrieval	I × E	≥6
Yokoo et al., 2021 [49]	sc-365501, mouse monoclonal, Santa Cruz Biotechnology	NR, Nichirei Biosciences	I + E	≥4
Hiltunen et al., 2022 [48]	sc-365501, mouse monoclonal, Santa Cruz Biotechnology	Dako EnVision^TM^ FLEX Target Retrieval	I + E	0–2
Sun et al., 2022 [73]	AB90582, rabbit polyclonal, Abcam	Dako EnVision^TM^ FLEX Target Retrieval	I	≥0.8
Zhao et al., 2022 [64]	20139-1-AP, rabbit polyclonal, ProteinTech Group Inc.	NR	IOD × E	>120

E, expression; I, intensity; IOD, integrated optical density; MC, membrane completeness; NR, not reported.

**Table 3:** Quality assessment of selected studies based on the guidelines from the Critical Appraisal Skills Programme (CASP) checklist for cohort studies [40].

Reference	Critical Appraisal Skills Programme (CASP) Checklist													Score	Classification
	1	2	4	5a	5b	6a	6b	7	8	9	10	11	12		
de Oliveira et al., 2012 [51]	●	●	●	●	●	●	●	●	●	●	●	●	●	17	Moderate
Choi et al., 2014 [74]	●	●	●	●	●	●	●	●	●	●	●	●	●	22	High
Eilertsen et al., 2014 [69]	●	●	●	●	●	●	●	●	●	●	●	●	●	22	High
Pértega-Gomes et al., 2014 [57]	●	●	●	●	●	●	●	●	●	●	●	●	●	17	Moderate
Pinheiro et al., 2014 [45]	●	●	●	●	●	●	●	●	●	●	●	●	●	19	Moderate
Polanski et al., 2014 [35]	●	●	●	●	●	●	●	●	●	●	●	●	●	21	High
Zhao et al., 2014 [30]	●	●	●	●	●	●	●	●	●	●	●	●	●	12	Low
Afonso et al., 2015 [27]	●	●	●	●	●	●	●	●	●	●	●	●	●	21	High
Andersen et al., 2015 [56]	●	●	●	●	●	●	●	●	●	●	●	●	●	19	Moderate
Kim et al., 2015 [60]	●	●	●	●	●	●	●	●	●	●	●	●	●	22	High
Afonso et al., 2016 [76]	●	●	●	●	●	●	●	●	●	●	●	●	●	22	High
Martins et al., 2016 [59]	●	●	●	●	●	●	●	●	●	●	●	●	●	23	High
Pinheiro et al., 2016 [41]	●	●	●	●	●	●	●	●	●	●	●	●	●	21	High
Pinheiro et al., 2016 [50]	●	●	●	●	●	●	●	●	●	●	●	●	●	21	High
Sousa-Simões et al., 2016 [42]	●	●	●	●	●	●	●	●	●	●	●	●	●	19	Moderate
Giatromanolaki et al., 2017 [70]	●	●	●	●	●	●	●	●	●	●	●	●	●	18	Moderate
Johnson et al., 2017 [71]	●	●	●	●	●	●	●	●	●	●	●	●	●	21	High
Latif et al., 2017 [43]	●	●	●	●	●	●	●	●	●	●	●	●	●	23	High
Mikkilineni et al., 2017 [53]	●	●	●	●	●	●	●	●	●	●	●	●	●	15	Moderate
Sáenz-de-Santa-María et al., 2017 [54]	●	●	●	●	●	●	●	●	●	●	●	●	●	18	Moderate
Wang et al., 2017 [67]	●	●	●	●	●	●	●	●	●	●	●	●	●	14	Moderate
Cao et al., 2018 [61]	●	●	●	●	●	●	●	●	●	●	●	●	●	20	Moderate
Li et al., 2018 [72]	●	●	●	●	●	●	●	●	●	●	●	●	●	21	High
Roseweir et al., 2018 [58]	●	●	●	●	●	●	●	●	●	●	●	●	●	21	High
Silva et al., 2018 [44]	●	●	●	●	●	●	●	●	●	●	●	●	●	21	High
Zhang et al., 2018 [75]	●	●	●	●	●	●	●	●	●	●	●	●	●	22	High
Afonso et al., 2019 [65]	●	●	●	●	●	●	●	●	●	●	●	●	●	21	High
Chen et al., 2019 [62]	●	●	●	●	●	●	●	●	●	●	●	●	●	22	High
Sukeda et al., 2019 [47]	●	●	●	●	●	●	●	●	●	●	●	●	●	18	Moderate
Zheng et al., 2019 [63]	●	●	●	●	●	●	●	●	●	●	●	●	●	20	Moderate
Dell’Anno et al., 2020 [52]	●	●	●	●	●	●	●	●	●	●	●	●	●	12	Low
Bonatelli et al., 2021 [55]	●	●	●	●	●	●	●	●	●	●	●	●	●	22	High
Eskuri et al., 2021 [66]	●	●	●	●	●	●	●	●	●	●	●	●	●	22	High
Leu et al., 2021 [46]	●	●	●	●	●	●	●	●	●	●	●	●	●	21	High
Tong et al., 2021 [68]	●	●	●	●	●	●	●	●	●	●	●	●	●	21	High
Yokoo et al., 2021 [49]	●	●	●	●	●	●	●	●	●	●	●	●	●	19	Moderate
Hiltunen et al., 2022 [48]	●	●	●	●	●	●	●	●	●	●	●	●	●	18	Moderate
Sun et al., 2022 [73]	●	●	●	●	●	●	●	●	●	●	●	●	●	24	High
Zhao et al., 2022 [64]	●	●	●	●	●	●	●	●	●	●	●	●	●	20	Moderate

Score of individual parameters: ● 0 points; ● 1 point; ● 2 points. Final classification: ■ low, ≤13 points; ■ moderate, 14–20 points; ■ high, >21 points.

## Data Availability

No new data were created or analysed in this study. Data sharing is not applicable to this article.
